# mtROS Induced *via* TLR-2-SOCE Signaling Plays Proapoptotic and Bactericidal Role in *Mycobacterium fortuitum*-Infected Head Kidney Macrophages of *Clarias gariepinus*

**DOI:** 10.3389/fimmu.2021.748758

**Published:** 2021-12-20

**Authors:** Priyanka Dahiya, Md. Arafat Hussain, Shibnath Mazumder

**Affiliations:** ^1^ Immunobiology Laboratory, Department of Zoology, University of Delhi, Delhi, India; ^2^ Faculty of Life Sciences & Biotechnology, South Asian University, New Delhi, India

**Keywords:** *M. fortuitum*, head kidney macrophage, TLR-2, ER stress, SOCE, mtROS, apoptosis

## Abstract

The mechanisms underlying *Mycobacterium fortuitum*-induced mycobacteriosis remain unexplored. Using head kidney macrophages (HKM) from catfish (*Clarias gariepinus*), we report that Ca^2+^ surge across mitochondrial-Ca^2+^ uniporter (MICU), and consequent mitochondrial ROS (mtROS) production, is imperative for mycobactericidal activity. Inhibition of mtROS alleviated HKM apoptosis and enhanced bacterial survival. Based on RNA interference (RNAi) and inhibitor studies, we demonstrate that the Toll-like receptor (TLR)-2–endoplasmic reticulum (ER) stress–store-operated calcium entry (SOCE) axis is instrumental for activating the mt-Ca^2+^/mtROS cascade in *M. fortuitum*-infected HKM. Additionally, pharmacological inhibition of mtROS attenuated the expression of *CHOP*, *STIM1*, and *Orai1*, which suggests a positive feedback loop between ER-stress-induced SOCE and mtROS production. Elevated tumor necrosis factor alpha (TNF-α) levels and caspase-8 activity were observed in HKM consequent to *M. fortuitum* infection, and our results implicate that mtROS is crucial in activating the TNF-mediated caspase-8 activation. Our results for the first time demonstrate mitochondria as an innate immune signaling center regulating mycobacteriosis in fish. We conclude that *M. fortuitum-*induced persistent SOCE signaling leads to mtROS production, which in turn activates the TNF-α/caspase-8 axis culminating in HKM apoptosis and bacterial clearance.

## Introduction

*Mycobacterium fortuitum*, atypical, rapidly growing, acid-fast mycobacteria, is one of the causative agents of mycobacteriosis. The occurrence of multidrug-resistant strains (1) along with its impact on aquaculture and zoonosis (2) makes it a pathogen of concern. Incidences of *M. fortuitum* infections in humans have also been reported (3). Even though the bacterium is known to infect a diverse range of hosts, there are very few reports that detail the molecular mechanisms of *M. fortuitum*-induced pathogenesis.

Toll-like receptors (TLRs) are a class of pathogen recognition receptors that recognize conserved molecular patterns expressed by pathogens triggering innate immune responses and inducing subsequent adaptive immune responses (4). Several TLRs have been reported to play critical roles in host immunity to mycobacterial pathogenesis, among which the involvement of TLR-2 is well studied (5, 6). The role of TLR-2 in mycobacterial immunity is contentious. It was observed that TLR-2 signaling contributes to mycobacterial immunity by secreting antibacterial molecules and proinflammatory cytokines, which recruit various immune effector cells to the site of infection (1, 7). TLR-2 knockout mice are more susceptible to *M. tuberculosis*, implicating the importance of TLR-2 signaling in mycobacterial immunity. Conversely, there are also reports suggesting that virulent mycobacteria utilize the TLR-2-MyD88 pathway for escaping from the phagosome and replicating in the cytosol (8). Prolonged stimulation of TLRs by persistent *M. tuberculosis* or its components render macrophages unresponsive to interferon gamma (IFN-γ) besides downregulating major histocompatibility complex (MHC) II expression and stimulating anti-inflammatory IL-10, thereby skewing the immune responses towards the pro-mycobacterial TH2 pole. Granuloma formation plays a major role in mycobacterial pathology, and it has also been observed that mycobacteria exploit TLR-2 signaling for facilitating granuloma formation and in the activation of peroxisome proliferator-activated receptor, which regulates lipid droplet accumulation inside macrophages together aiding in its survival in the host (9). Based on these studies, it is quite evident that the cross-talk between mycobacteria and TLR-2 has a profound impact in modulating host immunity and establishing chronicity in the host.

TLR-2 signaling impacts several downstream molecules including Ca^2+^, in response to bacterial pathogens (10, 11) and triggers ER stress; a condition characterized by depletion of Ca^2+^ inside ER-lumen and enhanced phosphorylation of eukaryotic translation initiation factor 2α (eIF2α), expression of glucose-regulated protein 78′ (GRP78 or BiP) and CCAAT/enhancer-binding homologous protein (CHOP) (8). Protracted ER stress triggers apoptosis, and CHOP plays a major role in this process (12). The role of ER stress in mycobacterial pathogenesis is well documented. It has been observed that mycobacteria-induced ER-stress results in macrophage apoptosis helping in the elimination of intracellular bacteria (11, 13). Previously, we had reported that TLR-2 plays an important role in the recognition and phagocytosis of mycobacteria by head kidney macrophages (HKM) (11). Fish possess a well-developed immune system that comprises both innate and adaptive components. The fish immune system exhibits remarkable resemblance with the mammalian immune system (14)m and the fish model has been successfully used to unravel molecular pathogenesis and immunology of several diseases including tuberculosis (15). The head kidney is a major immunocompetent organ in fish that houses different immune cell types including macrophages.

Recently, we observed that TLR-2 impacts cytosolic-Ca^2+^ [(Ca^2+^)_c_] levels by altering the expression of store operated calcium entry (SOCE) channels (16). Complementing this, TLR-2 activation has also been demonstrated to trigger mtROS production against *Salmonella typhimurium* by mammalian macrophages (7). However, the involvement of TLR-2 in modulating Ca^2+^ entry inside the mitochondrial matrix and subsequent mtROS production has not been explored in *M. fortuitum* infection.

SOCE, consisting of two key proteins, stromal interaction molecule 1 (STIM1) and ORAI calcium release-activated calcium modulator1 (Orai1), gets activated in response to ER stress and plays a critical role in maintaining long-term Ca^2+^ signals in addition to the replenishment of ER-Ca^2+^ [(Ca^2+^)_ER_] stores (17). STIM1 is a type I transmembrane bi-functional protein localized on the ER membrane, which senses Ca^2+^ levels inside the ER lumen and activates Orai1 expression in response to ER-Ca^2+^ depletion (18). Orai1 is a membrane-spanning protein with four transmembrane helices localized on the plasma membrane (19), which mediate Ca^2+^ influx when activated by STIM1 (20). The involvement of TLR-2–ER stress–STIM1/Orai1 axis-dependent cytosolic (Ca^2+^)*_c_* surge in *M. fortuitum-*induced pathogenesis has been evidenced (16). However, reports indicating the participation of STIM1/Orai1 signaling in mitochondrial dysfunction are obscure in fish.

Mitochondria are major sites of ATP production through oxidative phosphorylation. Transfer of electrons through a series of enzymatic donors and acceptors leads to the reduction in O_2_ to water and the consequent production of ATP. However, leakage of electrons during the respiratory process induces superoxide anions, major contributors of mtROS (21). Under normal cellular conditions, mtROS is produced in small amounts, which play an important role in cellular signaling and homeostasis. However, sustained production of mtROS under stress creates an imbalance between oxidant generation and antioxidant systems of mitochondria. Increased levels of Ca^2+^ inside the mitochondrial lumen [(Ca^2+^)_m_] is a key factor for the production of mtROS (22). ER stress triggers transport of Ca^2+^ inside mitochondria (mt-Ca^2+^) through SOCE across the mitochondrial calcium uniporter (MICU) (23, 24), and buffering of Ca^2+^ inside mitochondria sustains Ca^2+^ influx by preventing Ca^2+^-dependent slow deactivation of STIM1–Orai1 complex (24, 25). mtROS has been linked to bactericidal activity, although the exact mechanism linking mtROS generation with innate immunity remains obscure (7, 26). Additionally, mtROS has also been reported to trigger apoptosis of innate immune cells (27, 28). However, the role of mtROS in *M. fortuitum* pathogenesis is not yet reported.

The proinflammatory cytokine TNF has a major role in mounting effective host immunity, and TLR-2 signaling plays a primal role in inducing its production (13). Interestingly, unlike mammals, several TNF orthologs, bearing the TNF family signature [LV]-x-[LIVM]-x3-G-[LIVMF]-Y-[LIMVMFY]2-x2-[QEKHL], have been reported in teleosts (29), suggesting the cytokine to be evolutionarily conserved. It has also been observed that several fish species possess multiple TNF-α isoforms (30). Both *in vitro* and *in vivo* studies have confirmed that the fish TNF-α isoforms confer proinflammatory effects (31, 32) and serve as prototype M1 macrophage markers in fish (33). The connection between TNF production and apoptosis of macrophages in response to mycobacterial infection along with reduction in the bacterial load has also been demonstrated (11, 13). There are also reports that suggest the involvement of SOCE in TNF production (34). However, the same has not been reported in *M. fortuitum-*induced pathogenesis.

Fish are the natural host for *M. fortuitum* (35). We have previously established HKM as an alternate model to study the molecular mechanisms of *M. fortuitum*-induced pathogenesis (16, 36, 37). Our study implicated the role of TLR-2/Myd88 signaling in regulating *M. fortuitum* pathogenesis. We also reported that TLR-2-induced (Ca^2+^)_c_ imbalance triggers ER stress and STIM1/Orai1 expression in infected HKM; the coordinated participation of STIM1-Orai1 and superoxide is critical in inducing NO-mediated apoptosis of HKM (16). In the present study, we investigated the role of STIM1/Orai1 signaling on mtROS generation consequent to *M. fortuitum* infection. We report that STIM1/Orai1 signaling augments mtROS production, which in turn incites TNF-α-mediated apoptosis of HKM and the clearance of *M. fortuitum.*

## Materials and Methods

### Bacterial Strain and Growth Conditions

*M. fortuitum* (Strain 993) was purchased from Microbial Type Culture Collection and Gene Bank (MTCC), Chandigarh, India. The strain is sensitive to amikacin and resistant to ampicillin as suggested by the antibiogram. For infection studies, bacteria were grown to mid-log phase (120 h) in Middlebrook 7H9 broth (HiMedia), supplemented with 0.05% Tween-80, 0.50% glycerol, and 100 μg/ml ampicillin in a shaking incubator (120 rpm) at 30°C. Before infection, *M. fortuitum* clumping was removed by repeatedly passaging through a 26-G needle. Stocks were maintained at −80°C in 10% glycerol and in Lowenstein Jensen media (HiMedia) at 4°C for further use.

### Fish Maintenance

Catfish, *Clarias gariepinus* (Siluriformes: Clariidae, 100–150 g), were procured from local fish farms and maintained in 50-L glass tanks (2–3 fish per tank) under natural photoperiod. Prior to initiating the study, fish were acclimatized to laboratory conditions for 15 days, and fish health was monitored at regular intervals by morphological and pathological examinations (38).

### Isolation of HKM and Infection Studies

Head kidneys were aseptically removed and placed in Roswell Park Memorial Institute (RPMI)-1640 (Gibco-Invitrogen) with phenol-red indicator supplemented with 25 mM HEPES (Gibco-Invitrogen) containing 1% penicillin–streptomycin. Single-cell suspensions of each pair of head kidney were prepared using 100-mm wire mesh. The cell suspension was centrifuged at 400*×g* for 10 min at 4°C, the supernatant was discarded and the pellet resuspended and then layered on a discontinuous Percoll density gradient (34/51) and centrifuged at 400*×g* for 20 min at 4°C. The phagocyte-rich fraction appearing above the 34/51 interface was collected, washed, and incubated overnight at 30°C under 5% CO_2_ for adherence to sterile Petri dishes (Nunc). The non-adherent cells were removed carefully, and the adherent macrophages were obtained by incubation with 1% cell dissociation medium (59418C, Sigma) at 30°C for 20 min. The purity of the HKM was checked by staining with Wright Giemsa Stain (>90% pure), and viability was determined using 0.4% trypan blue dye exclusion method (>95% viable) (39, 40).

For infection, the HKM were washed in antibiotic-free RPMI supplemented with 10% fetal bovine serum (FBS) (Gibco-Invitrogen) and infected with *M. fortuitum* at a multiplicity of infection (MOI) of 1:10 (HKM/bacteria). The number of HKM used for different experiments is mentioned in the corresponding section. A short spin of 5 min was given to facilitate bacteria–HKM interactions, the cells were distributed in six-well tissue culture plates and incubated for 4 h at 30°C. Subsequently, amikacin (50 μg/ml, HiMedia) was added and the cells further incubated for 1 h to kill the extracellular bacteria. The concentration of amikacin effectively killed extracellular bacteria without affecting HKM viability (data not shown). Finally, the infected HKM were washed and resuspended in RPMI supplemented with 10% FBS containing amikacin (5 μg/ml) and incubated at 30°C for further studies (36, 37).

### Reagents

TLR-2 inhibitor (CUCPT-22, 1 μM), ER-stress inhibitor (4-phenyl butyric acid, 4-PBA, 10 µM), mtROS inhibitor (YCG063, 10 µM), mtROS inducer (antimycin A, Ant A, 50 µM), TNF-α biosynthesis inhibitor pentoxifylline (Pentox, 1 mM), and mt-Ca^2+^ uniporter blocker (Ru360, 10 µM) were purchased from Sigma. Caspase-8 inhibitor (Z-IETD-FMK, 10 µM) was purchased from Biovision. (Ca^2+^)*_c_* monitoring dye (Fluo-3/AM, 2 µM) and mt-Ca^2+^ monitoring dye (Rhod-2/AM, 5 µM) were purchased from Invitrogen. Mitochondrial superoxide indicator (Mitosox, 5 μM) was purchased from Molecular Probes. HKM were pretreated with specific inhibitors for 1 h prior to infection with *M. fortuitum*. The doses of different inhibitors were selected on the basis of inhibitor specificity and cytotoxicity. The HKM treated with the indicated concentrations of the inhibitors remained as viable as control HKM at all-time points as determined by the trypan blue (0.4%) dye exclusion method and were maintained during the entire course of the experiment (16, 36, 37).

### RNA Isolation, cDNA Synthesis, and Real-Time qPCR

HKM (2 × 10^7^/ml) pretreated with or without inhibitors or transfected with scrambled (sc-) or specific small-interfering RNAs (siRNAs) were harvested at indicated time points p.i., total RNA isolated using TRIZOL (Sigma), and dissolved in diethyl pyrocarbonate (DEPC) water. One microgram RNA was used as a template using first-strand complementary DNA (cDNA) synthesis kit (MBI fermentas). Primers for *CHOP*, *STIM1*, *Orai1*, *TNF-*, and *β-actin* genes were already available in our laboratory (**Table 1**). Fold change in *CHOP*, *STIM1*, *Orai1*, and *TNF* mRNA expression levels were studied using SYBR green PCR Master Mix (Applied Biosystems) by RT-qPCR (ABI ViiA, Applied Biosystems). cDNA (1:100 dilution), forward and reverse primers (0.20 µM each), and 5 µl SYBR green PCR master mix (Applied Biosystems) were used (total volume, 10 μl) for each assay. Expression levels of different genes were analyzed by the comparative ΔΔCT method wherein *β-actin* was taken as the endogenous control and uninfected HKM (0 h) was used as the calibrator (16).

**Table 1:** Real-time primer sequences.

Gene	Accession no.	Real-time primers
***STIM1***	**KU962938**	**FP** 5’-TGGGCCAGATGATGAAAGACC-3’
		**RP** 5’-CACCTTTTCCACCTCCACTGA-3’
***Orai1***	**KX765881**	**FP** 5’-CTCTGCTGGGTCAAGTTCCT-3’
		**RP** 5’-ACGATGATGCAGGTGGAGG-3’
***CHOP***	**LK054407**	**FP** 5’-GTTGGAGGCGTGGTATGAAG-3’
		**RP** 5’-GAAACTCCGGCTCTTTCTCG-3’
***TNF-α***	**KM593875**	**FP** 5’- TCTCAGGTCAATA- CAACCCGC-3’
		**RP** 5’-GAGGCCTTTGCGGAAAATCTTG -3’
***β-actin***	**AF057040**	**FP** 5’-CGAGCAGGAGATGGGAACC-3’
		**RP** 5’-CAACGGAAACGCTCATTGC-3’

### siRNA Transfection

siRNA (**Table 2**) transfection was done using HiPerFect Transfection Reagent (Qiagen). Briefly, 5 μl each of siRNA and HiPerFect complex were mixed gently, added to 90 μl Opti-Mem (Invitrogen), and incubated for 20 min at 30°C to allow complex formation. The complex was then added to the HKM cultures maintained in Opti-MEM, mixed properly (total volume, 1 ml), and incubated at 30°C with 5% CO_2_ for 16 h during which HKM viability was continuously monitored. Thereafter, HKM were infected with *M. fortuitum* and processed for subsequent studies. Targeted gene knockdown was confirmed by real-time quantitative PCR (RT-qPCR) using target-specific siRNAs. Scrambled or sc-siRNA (Sigma, 5 nM) was used as universal negative control in this study.

**Table 2:** siRNA sequences.

Gene	siRNA’s
***STIM1***	**Sense** 5’-GGGACCACAUGGGCCAGAUdTdT-3’
	**Anti-sense** 5’-AUCUGGCCCAUGUGGUCCCdTdT-3’
***Orai1***	**Sense** 5’-GCCUACGCCUCCACCUGCAdTdT-3’
	**Anti-sense** 5’-UGCAGGUGGAGGCGUAGGCdTdT-3’
***CHOP***	**Sense** 5’-AUGAAGACUUGCAAGAUAU- 3’
	**Anti-sense *5’-*** AUAUCUUGCAAGUCUUCAU-3’
***TNF-α***	**Sense** 5’-GCAAAGGCCUCUACUUCGU-3’
	**Anti-sense *5’-*** ACGAAGUAGAGGCC UUUGC-3’

### Measurement of mt-Ca^2+^

HKM (2 × 10^6^/ml) pretreated with or without inhibitors or transfected with sc- or targeted siRNAs were incubated with cell-permeable mt-Ca^2+^ dye Rhod-2/AM for 20 min at 30°C under dark conditions. Excess dye was removed by washing with phosphate-buffered saline (PBS) (1) and HKM infected with *M. fortuitum.* The changes in fluorescence intensity were measured at indicated time points p.i. in a fluorimeter (Spectramax, Molecular Devices) at excitation–emission of A_552_ and A_581_, respectively, and changes in mt-Ca^2+^ levels were plotted as the relative increase in fluorescence values.

### Measurement of mtROS

HKM (2 × 10^6^/ml) pretreated with or without inhibitors or transfected with sc- or targeted siRNAs were infected with *M*. *fortuitum*. HKM were collected at indicated time point p.i. and incubated with MitoSOX for 20 min at room temperature under dark conditions. Excess dye was removed by washing with PBS (1), and the changes in fluorescence intensity were measured at excitation–emission of A_510_ and A_580_, respectively (Spectramax, Molecular Devices). The changes in mtROS levels were plotted as the relative increase in fluorescence values.

### TNF-α Quantification

HKM (2 × 10^6^/ml) pretreated with or without inhibitors or transfected with sc- or targeted siRNAs were infected with *M*. *fortuitum*. Cell-free culture supernatant was collected at indicated time points p.i. and TNF-α levels measured with fish-specific TNF-α ELISA kit (MyBioSource, MBS704369). Briefly, 100 μl of culture supernatant was loaded to the antibody precoated wells and incubated at 37°C for 90 min. The supernatant was removed, and 100 μl biotinylated detection antibody was added followed by incubation at 37°C for 1 h. Wells were washed and 100 μl horseradish peroxidase (HRP) conjugate was added, and the plates were incubated at 37°C for 30 min. Following incubation, the wells were washed, and 90 μl substrate was added and incubated at 37°C for 15 min. Fifty microliters of stop solution was added to terminate the reaction and absorbance read at A_450_ (Spectramax, Molecular Devices). The concentration of TNF-α in each sample was interpolated from the standard curve (11).

### Enumeration of Intracellular Bacteria

HKM (2 × 10^6^/ml) pretreated with specific inhibitors or transfected with sc-siRNA or targeted siRNAs, respectively, were infected with *M. fortuitum*. The cultures were terminated at indicated time point p.i., lysed with 0.1% Triton X-100, serially diluted, plated on 7 H11 Middlebrook agar plates supplemented with 0.05% Tween-80, 0.50% glycerol, and 100 μg/ml ampicillin. Intracellular bacteria (CFU) were enumerated following incubation at 30°C (16).

### Caspase Assay

HKM (2 × 10^6^/ml) pretreated with or without specific inhibitors or transfected with sc-siRNA or targeted siRNAs were infected with *M. fortuitum*. Caspase-8 (Elabscience) and caspase-3 activity (Biovision) were studied using specific assay kits. Briefly, HKM collected at indicated time points p.i. were lysed, and to the cell lysate (50 μl), equal volume of 2 reaction buffer and caspase-8/caspase-3-specific substrate (5 μl) was added to separate wells and mixed gently, avoiding bubble formation. The plates were incubated at 37°C for 2 h, absorbance was measured at A_405_ (Spectramax, Molecular Devices), and relative fold changes in caspase-8 and caspase-3 activity were plotted.

### Statistical Analysis

Mean ± SE were calculated using pairwise comparison by employing *t*-test: two samples using unequal variances to determine the statistical significance between the groups. A value of *p* < 0.05 was considered as statistically significant. Individual assays were done in triplicates, and the vertical bars represent mean ± SE of three independent observations (n = 9).

### *M. fortuitum-*Induced mt-Ca^2+^ Surge Triggers mtROS in HKM

**Figure 1:**
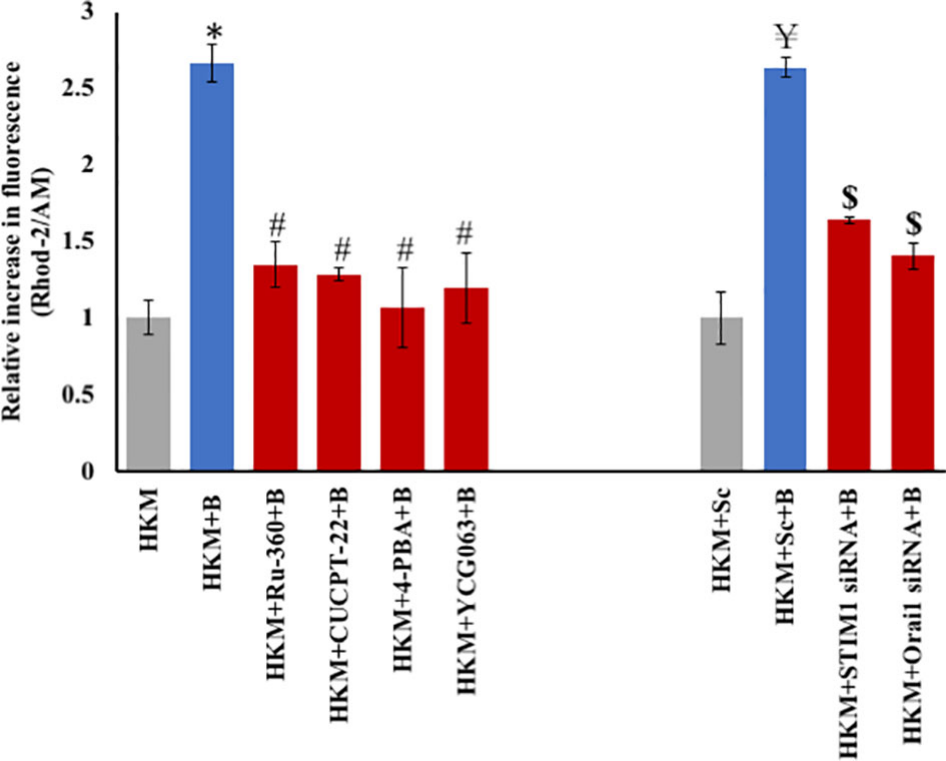
*M. fortuitum-*induced SOCE triggers mt-Ca^2+^ elevation. HKM (2 × 10^6^/ml) pretreated separately with or without specific inhibitor or transfected with sc-siRNA or targeted siRNA were infected with M*. fortuitum* and mt-Ca^2+^ elevation measured at 2 h p.i. using Rhod-2/AM. Individual assays were done in triplicates, and the vertical bars represent mean ± SE of three independent observation (n = 9). ^*^ *p* < 0.05 compared to HKM; *^#^p* < 0.05 compared to HKM+B; *^￥^p* < 0.05 compared to HKM+Sc; *^$^p* < 0.05 compared to HKM+Sc+B. HKM, uninfected HKM; HKM+B, HKM infected with *M. fortuitum*; HKM+Ru-360+B, HKM+CUCPT-22+B, HKM+4-PBA+B, HKM+YCG063+B, HKM pretreated with CUCPT-22, 4-PBA, Ru-360, YCG063, respectively, and infected with *M. fortuitum*. HKM+Sc, HKM transfected with sc-siRNA; HKM+Sc+B, HKM transfected with sc-siRNA and infected with *M. fortuitum*; HKM+STIM1 siRNA+B; HKM+Orai1 siRNA+B, HKM transfected with STIM1 and Orai1 siRNA, respectively, and infected with *M. fortuitum* (Ru-360, MICU inhibitor; CUCPT-22, TLR-2 inhibitor; 4-PBA, ER-stress inhibitor; YCG063, mtROS inhibitor).

The role of mt-Ca^2+^-induced mtROS as an innate immune factor has been reported previously (7, 41). In absence of prior knowledge, our aim was to study this in *M. fortuitum* pathogenesis. HKM were infected with *M. fortuitum*, and mt-Ca^2+^ levels were monitored at indicated time points p.i. using mt-Ca^2+^-specific dye Rhod-2/AM. We observed a significant increase in mt-Ca^2+^ levels with maximum levels recorded at 2 h p.i. (**Supplementary Figure 1**), and selected this time interval for subsequent studies. The mitochondrial uniporter MICU mediates the influx of (Ca^2+^)*_c_* inside mitochondria, triggering mtROS production (42). To look into this, HKM were pretreated with MICU inhibitor Ru360 and then infected with *M. fortuitum*, and mt-Ca^2+^ levels were monitored at 2 h p.i. It was observed that mt-Ca^2+^ levels were significantly downregulated in the presence of Ru360 (**Figure 1**), suggesting the involvement of MICU in the dynamics of mt-Ca^2+^ in *M. fortuitum* infection. Furthermore, we pre-treated the HKM with TLR-2 and ER-stress inhibitors (CUCPT-22 and 4-PBA) followed by infection with *M. fortuitum* and measured the mt-Ca^2+^ levels (**Figure 1**). Our results affirmed the involvement of TLR-2-ER-stress-STIM1-Orai1 axis in triggering mt-Ca^2+^ elevation p.i. Additionally, we also measured mt-Ca^2+^ levels in the absence of STIM1 and Orai1 signaling. For that, the HKM were transfected with STIM1 and Orai1 siRNA, respectively, and then infected with *M. fortuitum* and the changes in mt-Ca^2+^ levels monitored. The significant reduction in mt-Ca^2+^ levels led us to conclude the involvement of SOCE in triggering mt-Ca^2+^ influx in *M. fortuitum*-infected HKM (**Figure 1**).

**Figure 2:**
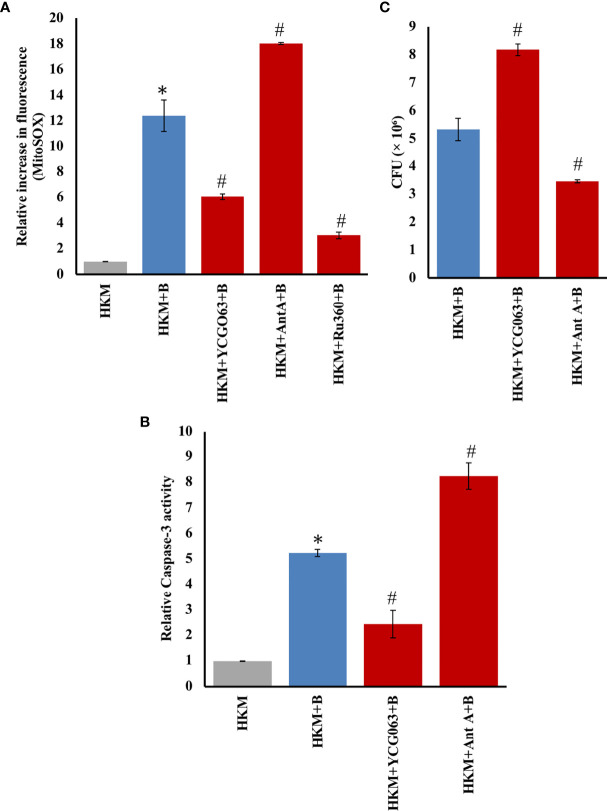
mtROS triggers apoptosis of *M. fortuitum-*infected HKM. HKM (2 × 10^6^/ml) pretreated separately with or without specific inhibitors were infected with *M. fortuitum* and **(A)** mtROS levels measured at 4 h p.i. using MitoSOX and **(B)** caspase-3 activity monitored at 24 h p.i. **(C)** HKM (2 × 10^6^/ml) were pretreated with or without YCG063 or Ant A separately and infected with *M. fortuitum* and the bacterial uptake determined by CFU at 24 h p.i. Individual assays were done in triplicates, and the vertical bars represent mean ± SE of three independent observations (n = 9). ^*^p < 0.05 compared to HKM; *^#^p* < 0.05 compared to HKM+B. HKM, uninfected HKM; HKM+B, HKM infected with *M. fortuitum*; HKM+YCG063+B, HKM+Ant A+B, HKM+Ru360+B, HKM pretreated with YCG063, Ant A, Ru360, respectively, and infected with *M. fortuitum* (YCG063, mtROS inhibitor; Ant A, mtROS inducer; Ru360, MICU inhibitor).

Our next step was establishing the link between mt-Ca^2+^ dynamics and mtROS production. To study this, we used the specific dye MitoSOX (43). *Mycobacterium fortuitum*-infected HKM were stained with MitoSOX, and the changes in mtROS levels were monitored at indicated time points p.i. We observed maximum mtROS levels at 4 h p.i. (**Supplementary Figure 2**) and selected this time interval for subsequent studies. In our next step, we pretreated the HKM with Ru360 and measured mtROS in infected HKM. We observed a significant reduction in mtROS levels in the presence of Ru360 (**Figure 2A**). Based on our kinetics and inhibitor studies, it is evident that the influx of mt-Ca^2+^ through MICU leads to downstream mtROS generation in *M. fortuitum*-infected HKM. Additionally, we also checked whether inhibition of mtROS is having any impact on the mt-Ca^2+^ levels. To this, we pretreated the HKM with mtROS inhibitor (YCG063) and measured the mt-Ca^2+^ levels. Our results suggested the existence of a positive feedback loop between mt-Ca^2+^ and mtROS elevation (**Figure 1**).

### mtROS Is Proapoptotic and Mycobactericidal

mtROS is reported to trigger apoptosis of host immune cells (44). However, the same is not well reported in fish. Our previous studies demonstrated that *M. fortuitum* induces caspase-3-dependent HKM apoptosis (36). Here, we hypothesized that mtROS generated in response to *M. fortuitum* infection induces HKM apoptosis. To begin with, HKM were pretreated with the mtROS inhibitor YCG063 (45) and then infected with *M. fortuitum* and mtROS levels monitored at 4 h p.i. We noted that pretreatment with YCG063 significantly downregulated mtROS levels in infected HKM (**Figure 2A**). Next, HKM pretreated with YCG063 were infected with *M. fortuitum* and apoptosis studied by caspase-3 assay at 24 h p.i. Pretreatment with YCG063 attenuated caspase-3 activity (**Figure 2B**). Antimycin A (Ant A), an inhibitor of complex III of the ETC, was used as positive control, which very predictably led to the production of measurable quantities of mtROS in HKM (**Figure 2A**) and triggered caspase-3 activity (**Figure 2B**). Thus, our results confirmed that mtROS plays a proapoptotic role in *M. fortuitum* pathogenesis.

We concluded investigating the role of mtROS in regulating *M. fortuitum* growth. HKM pretreated with YCG063 were infected with *M. fortuitum*, and the growth of intracellular bacteria was studied at 24 h p.i. We observed that inhibiting mtROS production by YCG063 led to a significant increase in the number of intracellular *M. fortuitum* (**Figure 2C**). Furthermore, pretreatment with mtROS-inducer Ant A also resulted in a significant reduction in the number of intracellular *M. fortuitum* (**Figure 2C**). Collectively, our results implicate that mtROS induces HKM apoptosis and helps in the clearance of *M. fortuitum*.

### The Crosstalk Between TLR-2–ER Stress–SOCE Axis and mtROS Is a Key Event in *M. fortuitum* Pathogenesis

**Figure 3:**
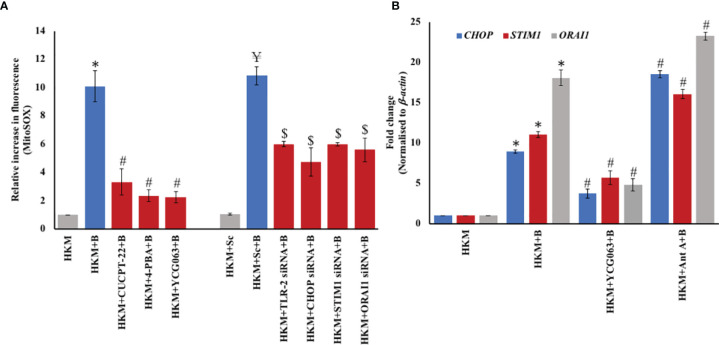
TLR-2–ER stress–SOCE axis triggers mtROS production and *vice versa*. **(A)** HKM (2 × 10^6^/ml) pretreated separately with or without specific inhibitors or transfected with scrambled siRNA (sc-siRNA) or targeted siRNAs, respectively, were infected with *M. fortuitum* and mtROS levels measured at 4 h p.i. using MitoSOX. **(B)** HKM (2 × 10^7^/ml) pretreated separately with or without specific inhibitors were infected with *M. fortuitum* and *CHOP*, *STIM1*, and *Orai1* mRNA expression quantified by RT-qPCR at 2 hr p.i. using SYBR green PCR master mix. Individual assays were done in triplicates, and the vertical bars represent mean ± SE of three independent observation (n = 9). ^*^p < 0.05 compared to HKM; *^#^P* < 0.05 compared to HKM+B; *^¥^P* < 0.05 compared to HKM+Sc; *^$^p* < 0.05 compared to HKM+Sc+B. HKM, uninfected HKM; HKM+B, HKM infected with *M. fortuitum*; HKM+CUCPT-22+B, HKM+4-PBA+B, HKM+YCG063+B, HKM+Ant A+B, HKM pretreated with CUCPT-22, 4-PBA, YCG063, Ant A, respectively, and infected with *M. fortuitum*; HKM+Sc, HKM transfected with sc-siRNA; HKM+TLR-2 siRNA+B, HKM+CHOP siRNA+B, HKM+STIM1 siRNA+B, HKM+Orai1 siRNA+B, HKM transfected with TLR-2, CHOP, STIM1, Orai1-siRNA, respectively, and infected with *M. fortuitum* (CUCPT-22, TLR-2 inhibitor; 4-PBA, ER-stress inhibitor; YCG063, mtROS inhibitor; Ant A, mtROS inducer).

We wanted to explore the upstream signaling events triggering mtROS production in infected HKM. Based on our own study (16) and previous reports (7), we hypothesized the primal role of the TLR-2-ER-stress axis in the process. To test this, HKM were treated separately with the TLR-2-specific inhibitor, CUCCPT-22 (46), or transfected with TLR-2-siRNA, then infected with *M. fortuitum*, and the changes in mtROS production (4 h p.i.) were monitored. We observed that inhibiting the TLR-2 signaling resulted in the downregulation of mtROS production in infected HKM. We followed this by pretreating the HKM with the ER-stress ameliorator, 4-PBA (47), or transfecting the HKM with CHOP-siRNA and monitoring the changes in mtROS levels. It was observed that inhibiting ER stress resulted in significant downregulation in the production of mtROS in *M. fortuitum*-infected HKM (**Figure 3A**). YCG063 was used as the control.

We had previously reported the proapoptotic role of SOCE in *M. fortuitum* pathogenesis (16). Here, we questioned the involvement of SOCE in mtROS generation in *M. fortuitum*-infected HKM. Towards that direction, we silenced *STIM1* and *Orai1* expression with specific siRNAs and measured mtROS levels in *M. fortuitum*-infected HKM. We observed that the silencing of *STIM1* and *Orai1* interfered with mtROS production in the infected HKM (**Figure 3A**). Based on these observations, we suggest that signaling *via* TLR-2–ER stress–SOCE axis induces mtROS generation in *M. fortuitum-*infected HKM.

We extended the study by pretreating HKM with the mtROS inhibitor, YCG063, and monitored the expression of *CHOP*, *STIM1*, and *Orai1*, respectively at 2 h p.i (16, 37). It was observed that inhibiting mtROS production downregulated *CHOP*, *STIM1*, and *Orai1* mRNA expression in *M. fortuitum*-infected HKM (**Figure 3B**). However, pretreatment with mtROS inducer Ant A resulted in a significant increase in *CHOP*, *STIM1*, and *Orai1* mRNA expression levels in *M. fortuitum*-infected HKM. To this, we concluded that the cross-talk between TLR-2–ER stress–SOCE axis and mtROS production potentiates *M. fortuitum-*induced HKM pathology.

### STIM1-Orai1/mtROS Crosstalk Triggers TNF-α Production in Infected HKM

mtROS is reported to induce the production of proinflammatory TNF-α with apoptotic implications (48). However, evidence of any link between ER stress–SOCE–mtDNA axis-dependent mtROS production and TNF-α synthesis remains unexplored to date. To begin with, HKM were infected with *M. fortuitum* and *TNF-α* mRNA expression studied at indicated time points p.i. We observed maximum *TNF-α* mRNA expression at 6 h p.i. (**Supplementary Figure 3A**). Complementing this, the changes in TNF-α protein levels were also studied using a specific assay kit. Maximum TNF-α production was observed at 24 h p.i. and selected for subsequent studies (**Supplementary Figure 3B**).

**Figure 4:**
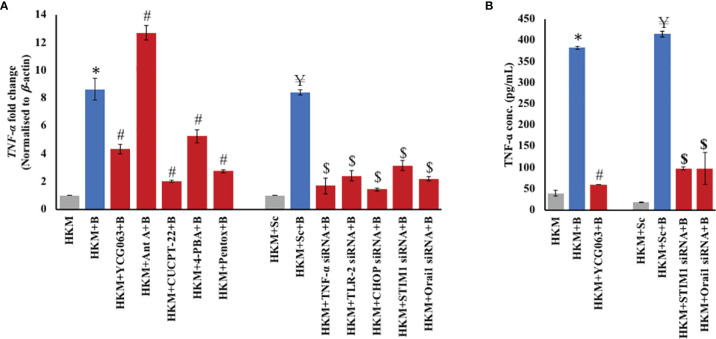
TLR-2–ER stress–STIM1/Orai1 signalosome induces TNF-α production. **(A)** HKM (2 × 10^7^/ml) pretreated separately with or without specific inhibitors or transfected with scrambled siRNA (sc-siRNA) or targeted siRNAs, respectively, were infected with *M. fortuitum* and *TNF-α* mRNA expression quantified by RT-qPCR at 6 h p.i. **(B)** HKM (2 × 10^6^/ml) pretreated separately with or without specific inhibitors or transfected with scrambled siRNA (sc-siRNA) or targeted siRNAs, respectively, were infected with *M. fortuitum* and TNF-α production was quantified at 24 h p.i. using specific assay kit. Individual assays were done in triplicates, and the vertical bars represent mean ± SE of three independent observations (n = 9). ^*^p < 0.05 compared to HKM; *^#^p* < 0.05 compared to HKM+B; *^¥^p* < 0.05 compared to HKM+Sc; *^$^p* < 0.05 compared to HKM+Sc+B. HKM, uninfected HKM; HKM+B, HKM infected with *M. fortuitum*; HKM+YCG063+B, HKM+Ant A+B, HKM+CUCPT-22+B, HKM+4-PBA+B, HKM+Pentox+B, HKM pretreated with YCG063, Ant A, CUCPT-22, 4-PBA, Pentox, respectively, and infected with *M. fortuitum*; HKM+Sc, HKM transfected with sc-siRNA; HKM+Sc+B, HKM transfected with sc-siRNA and infected with *M. fortuitum*; HKM+TLR-2 siRNA+B, HKM+CHOP siRNA+B, HKM+STIM1 siRNA+B, HKM+Orai1 siRNA+B, HKM transfected with TLR-2, CHOP, STIM1, Orai1-siRNA, respectively, and infected with *M. fortuitum* (YCG063, mtROS inhibitor; Ant A, mtROS inducer; CUCPT-22, TLR-2 inhibitor; 4-PBA, ER-stress inhibitor; Pentox, TNF-α inhibitor).

Next, HKM pretreated with YCG063 were infected with *M. fortuitum*, and changes in *TNF-α* mRNA expression and protein levels were monitored at 6 and 24 h p.i. respectively (**Figures 4A, B** **)**. We observed that inhibiting mtROS production resulted in a significant decline in TNF*-*α mRNA expression and protein concentration. In addition to this, treatment with Ant A led to significant upregulation in *TNF-α* mRNA expression, which led us to conclude that *M. fortuitum* infection in fish triggers mtROS-dependent TNF-α production.

We extended our study wherein HKM pretreated with CUCPT-22, 4-PBA, or transfected separately with *TLR-2-*, *CHOP-*, *STIM1-*, and *Orai1-*siRNA were infected with *M. fortuitum* and the changes in TNF-α levels monitored. It was observed that silencing of TLR-2 signaling, ameliorating ER stress, inhibiting STIM1/Orai1 expression, and mt-Ca^2+^ influx led to significant downregulation in TNF-α mRNA and protein expression (**Figures 4A, B** **)** in the infected HKM. Pentox was used as a control for the study. Collectively, these results implicated the critical role of mtROS in inducing TNF-α production in *M. fortuitum*-infected HKM and that TLR-2/ER stress/SOCE-mt-Ca^2+^ axis plays a primal role in the process.

### TNF-α Affects HKM Apoptosis and Clearance of *M. fortuitum*

**Figure 5:**
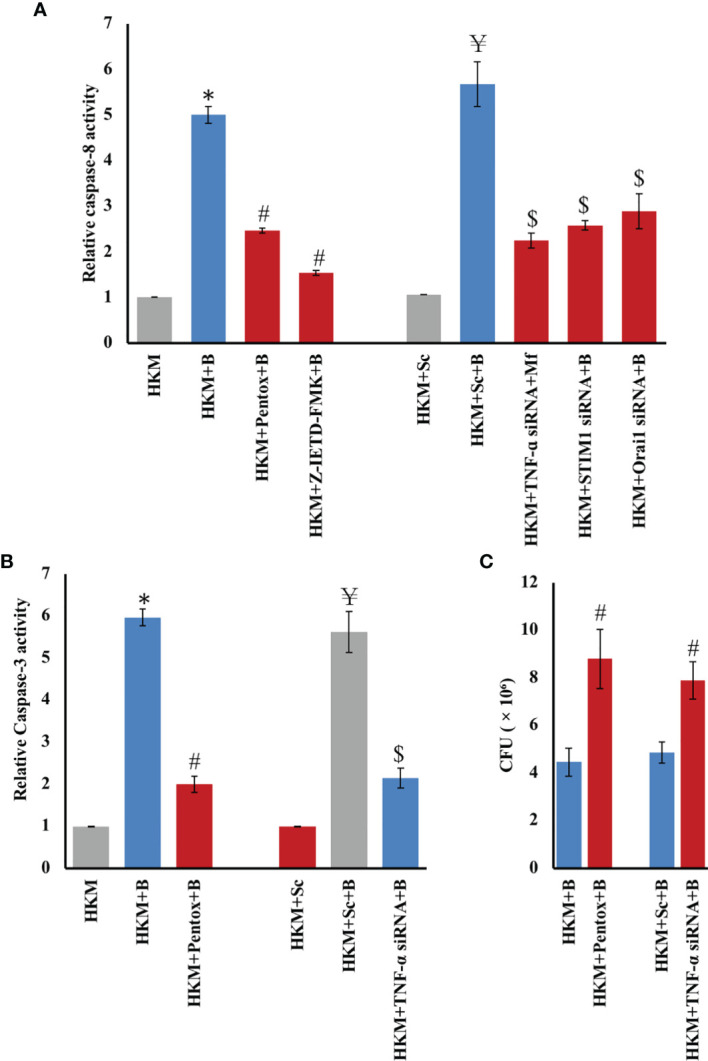
TNF-α/caspase-8 axis triggers caspase-3 mediated apoptosis and clearance of *M. fortuitum.* HKM (2 × 10^6^/mL) pre-treated separately with or without specific inhibitors or transfected with scrambled siRNA (sc-siRNA) or targeted siRNAs respectively were infected with *M. fortuitum* and **(A)** caspase-8 **(B)** caspase-3 activity monitored at 24 h p.i. using specific assay kit. **(C)** HKM (2 × 10^6^/mL) pre-treated separately with or without specific inhibitors or transfected with scrambled siRNA (sc-siRNA) or targeted siRNAs, respectively, were infected with *M. fortuitum* and bacterial uptake determined by CFU at 24 h p.i. Individual assays were done in triplicates, and the vertical bars represent mean ± SE of three independent observation (n = 9). ^*^p < 0.05 compared to HKM; *^#^p* < 0.05 compared to HKM+B; ^¥^ *p* < 0.05 compared to HKM+Sc; *^$^p* < 0.05 compared to HKM+Sc+B. HKM, uninfected HKM; HKM+B, HKM infected with *M. fortuitum*; HKM+Pentox+B, HKM+Z-IETD-FMK+B, HKM pre-treated with Pentox, Z-IETD-FMK, respectively, and infected with *M. fortuitum*; HKM+Sc, HKM transfected with sc-siRNA; HKM+Sc+B, HKM transfected with sc-siRNA and infected with *M. fortuitum*; HKM+ TNF-α siRNA+B HKM+STIM1 siRNA+B, HKM+Orai1 siRNA+B, HKM transfected with TNF-α, STIM1, Orai1-siRNA, respectively, and infected with *M. fortuitum* (Pentox, TNF-α inhibitor; Z-IETD-FMK, caspase-8 inhibitor).

TNF-α is well known to induce the activation of caspase-8 under various conditions of stress (49, 50). We had previously observed that *M*. *fortuitum* triggers caspase-8-mediated apoptosis of HKM (36). We questioned the role of TNF-α in activating caspase-8 in *M. fortuitum*-infected HKM. For that, HKM pretreated with TNF-α inhibitor pentox or transfected with TNF-α siRNA were infected with *M. fortuitum*, and the changes in caspase-8 levels were studied 24 h p. i (36). It was observed that inhibiting TNF-α led to a significant decrease in caspase-8 activity (**Figure 5A**), which clearly suggested that TNF triggers caspase-8 activation in *M. fortuitum*-infected HKM. Caspase-8 inhibitor Z-IETD-FMK was used as a control in the study.

Caspase-8 activation leads to both caspase-3-dependent and caspase-3-independent apoptosis (51). To study this, HKM pretreated with caspase-8 inhibitor Z-IETD-FMK were infected with *M. fortuitum*, and caspase-3 activity was monitored at 24 p.i (36). We observed that inhibiting caspase-8 activation resulted in significant downregulation in caspase-3 activity. Furthermore, pretreatment with pentox or transfection with TNF-α siRNA also markedly inhibited caspase-3 activation in *M. fortuitum*-infected HKM (**Figure 5B**). Based on these results, it is evident that the activation of the TNF-α/caspase-8 axis leads to caspase-3-mediated apoptosis of *M. fortuitum* **-**infected HKM.

Next, we investigated the role of TNF in deciding the fate of intracellular *M. fortuitum*. To achieve this, HKM pretreated with TNF inhibitor pentox and TNF siRNA were infected with *M. fortuitum* and intracellular bacteria (CFU) enumerated at 24 p.i. Inhibition of TNF production led to significant improvement in intracellular bacterial number (**Figure 5C**), implicating the bactericidal role of TNF in *M. fortuitum* pathogenesis. Taken together, our findings established that TNF-induced HKM apoptosis aids in the clearance of *M. fortuitum*.

## Discussion

*Mycobacterium fortuitum* induces apoptosis of host macrophages, but the underlying mechanisms remain unexplained. In the present study, we examined the role of mtROS in intracellular survival and pathogenesis of *M. fortuitum* using HKM as a model. Our present findings suggest mtROS as an important host innate immune attribute in regulating *M. fortuitum* pathogenesis.

Among several signaling molecules that regulate mycobacterial pathogenesis, Ca^2+^ is important (36, 52). Under stress, ER releases Ca^2+^, a significant portion of which enters the mitochondria through MICU. The balanced uptake of Ca^2+^ is crucial for mitochondrial metabolism, but the sustained accumulation of Ca^2+^ is detrimental for a cell. It amplifies the production of toxic mtROS (22), which interferes with mitochondrial functions, inducing apoptosis (53). We noted that *M. fortuitum* infection led to a significant rise in mt-Ca^2+^ levels, which coincided with heightened mtROS production, suggesting a positive correlation between mt-Ca^2+^ levels and mtROS production in infected HKM. Although mtROS generation has been observed in other mycobacteria (54, 55), this is the first report demonstrating *M. fortuitum* altering mt-Ca^2+^ dynamics triggering mtROS generation in infected macrophages. Our next step was studying the role of mtROS in *M. fortuitum* pathogenesis. We had earlier observed that inhibiting mtROS production restored mitochondrial membrane potential (37). Extending that in the present study, we noted that inhibiting mtROS production attenuated caspase-3 activity and interfered with HKM apoptosis, thereby favoring intracellular *M. fortuitum* growth, while augmenting mtROS production had the opposite effects, suggesting a role of mtROS in *M. fortuitum* pathology. The role of mtROS in mycobacterial pathogenesis is contentious. It has been reported that mtROS repress proinflammatory responses and facilitates the survival of *M. abscessus* in macrophages (55). mtROS has also been suggested to be a virulence attribute aiding in phagosome rupture and escape of mycobacteria to the cytosol where it replicates efficiently, triggering necrosis and spreading to adjacent cells (55, 56). Additionally, the implication of apoptosis in mycobacterial pathogenesis is also not clear (57, 58). Our results distinctly establish the proapoptotic and bactericidal role of mtROS in *M. fortuitum* infection. These findings are in accord with the bactericidal role of mtROS reported previously against several microbial pathogens (7, 59, 60). Based on these findings, we propose that mtROS generation is pro-host against *M. fortuitum* infection in fish. The ability to mount effective immunity against a particular pathogen is host centric, and at this stage, we are not sure whether this proapoptotic bactericidal role of mtROS is a fish-specific innate response against mycobacteria or conserved in other vertebrates, too. We previously recorded increased intracellular ROS in *M. fortuitum*-infected HKM (36). Extending our previous findings, we propose that mtROS contributes to total cellular ROS produced in infected HKM, thereby compounding *M. fortuitum* pathogenesis.

Identifying the upstream molecules that influence mtROS production in *M. fortuitum* infection was our next step. We recently demonstrated that TLR-2 augments (Ca^2+^)*c* surge triggering ER stress with proapoptotic implications in *M. fortuitum*-infected HKM (16). Here, we studied the involvement of TLR-2 in initiating mtROS axis and observed that in the absence of TLR-2 signaling, there was a marked reduction in mtROS levels. These results support previous studies suggesting that TLR-2 activation elicits mtROS generation in bacteria-infected cells (7, 59, 60). mtROS generation is intimately linked with the assembly and functioning of ETC. Previous studies have suggested that the interaction of TLR-2 adaptor molecule tumor necrosis factor receptor-associated factor 6 (TRAF-6) with evolutionarily conserved signaling intermediate in Toll pathways (ECSIT) impacts ETC assembly, triggering mtROS generation (7). Identifying the downstream adaptor molecules and kinases of TLR-2 cascade influencing mtROS generation in fish macrophages will help in understanding the molecular underpinnings of *M. fortuitum* pathogenesis and associated therapeutics. Based on these results, we extend our previous findings to suggest that besides functioning as an immune sensor, TLR-2 also aids in linking *M. fortuitum* stimuli with antibacterial mtROS generation. Our findings firmly establish the role of mitochondria in fish innate immunity and suggest that TLR-2 functions as a conduit between them.

Once we observed the primal role of TLR-2 in mtROS generation, we asked how TLR-2 induces mtROS during *M. fortuitum* infection. Several mechanisms have been proposed linking TLR-2 with mtROS generation under varying conditions of pathogen stress (7). Prolonged ER stress is harmful to cells, and our own findings suggested the role of TLR-2 in triggering prolonged ER-stress and mitochondrial dysfunction in mycobacteria-infected HKM (11, 37). The contribution of SOCE in activating the mt-Ca^2+^/mtROS axis has been reported (61). However, the role of SOCE in the alteration of mitochondrial homeostasis was not studied in mycobacterial infection. A comparable decline in mt-Ca^2+^ on inhibiting STIM1/Orai1 signaling led us to conclude that SOCE contributed towards the mt-Ca^2+^ surge in infected HKM. Our earlier studies with cytochalasin D, which inhibits mitochondrial movement (37) coupled with MICU inhibitor Ru-360 here, suggest persistent SOCE sustains mt-Ca^2+^ across MICU, and the temporal association between ER and mitochondria is critical for the transport of Ca^2+^ between the two organelles triggering mtROS generation. What determines mitochondrial movement propelling it towards ER in *M. fortuitum*-infected HKM is not clear from this study. Mitochondrial adaptor proteins act as Ca^2+^ sensors and play a major role in mitochondrial motility through their interactions with different motor proteins and cytoskeletal proteins (62). It has also been observed that mt-Ca^2+^ content *per se* influences mitochondrial mobility in neurons, and MICU regulates the process by gating Ca^2+^ influx into the organelle (63). Future studies aimed towards examining the mechanism by which mt-Ca^2+^ influx through MICU affects the interactions of mitochondrial adaptor proteins and motor proteins in infected HKM will help in understanding mitochondrial dynamics following *M. fortuitum* infection and ensuing pathogenesis induced by the bacterium.

ER stress and SOCE are intimately linked. Complementing this, we observed a positive correlation between mtROS and the expression of *CHOP*, *STIM1*, and *Orai1*, respectively. This finding suggests that a positive feedback loop is activated between ER-stress-dependent SOCE and mtROS production consequent to *M. fortuitum* infection. Since the three events are inherently cytotoxic in nature, we propose that the trio plays a non-redundant and complementary role in HKM apoptosis.

We intended to study how mtROS influences *M. fortuitum*-induced HKM apoptosis. Proinflammatory cytokines induce macrophage apoptosis, and mtROS has been reported to trigger the synthesis of proinflammatory cytokines (64). SOCE has also been linked to the synthesis of proinflammatory TNF-α (65). To the best of our knowledge, there are no reports that demonstrate the involvement of the SOCE–mtROS axis in inducing TNF-α synthesis in mycobacterial infection. The role of TNF-α is contentious in fish with reports suggesting that it mediates both susceptibility and resistance to mycobacterial pathogens (11, 66, 67). Additionally, the nature of cell death induced by TNF-α is debatable with reports suggesting its role both in apoptosis (11) and necrosis (67) of mycobacteria infected fish macrophages. TNF-α induces macrophage apoptosis by activating caspase-8 (68). Notably, the proapoptotic role of the TNF-α–caspase-8 axis is not well narrated in piscine mycobacteriosis. We observed that inhibition of TLR-2–ER stress–SOCE-mediated mtROS generation repressed TNF-α production in the infected HKM, which clearly established TNF-α activation as a downstream target of the axis. Furthermore, RNA interference (RNAi) and inhibitor studies demonstrated that inhibiting TNF-α interfered with caspase-8 activity, revoked HKM apoptosis, and aided *M. fortuitum* growth, which clearly suggested that (1) mtROS exerts its proapoptotic effects *via* TNF-α in *M. fortuitum*-infected HKM and (2) TNF-α-induced HKM apoptosis helps in pathogen clearance, thereby containing the persistence of *M. fortuitum* in fish.

**Figure 6:**
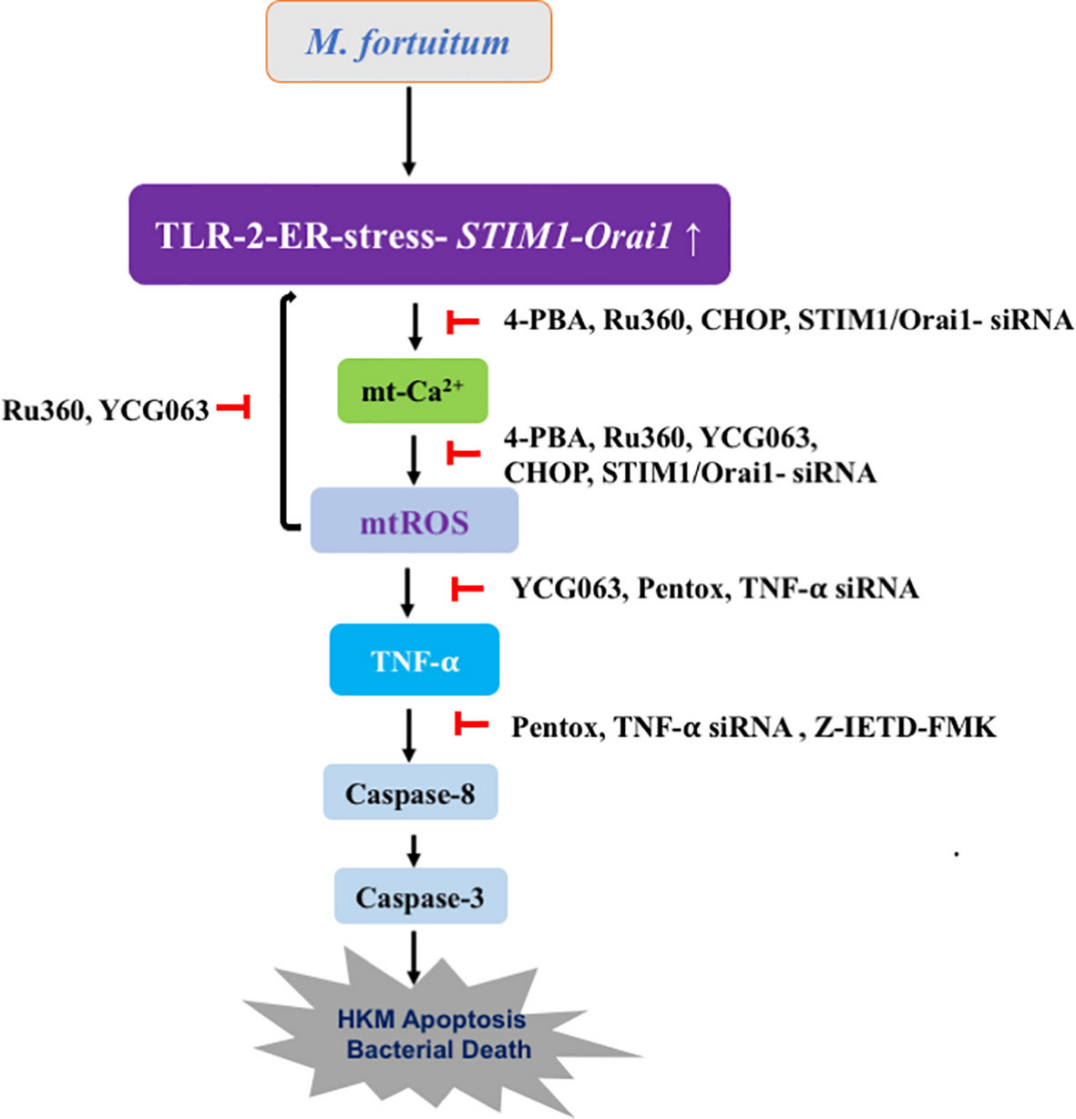
Overview of the work. TLR-2–ER stress–SOCE axis triggers mtROS production in *M. fortuitum*-infected HKM. Crosstalk between SOCE and mtROS amplifies TNF-α production, which culminates in caspase-8/3-dependent HKM apoptosis and *M. fortuitum* clearance.

To conclude, the TLR-2–ER stress–SOCE axis triggers mtROS generation in *M. fortuitum*-infected HKM. Furthermore, the crosstalk between SOCE and mtROS amplifies proinflammatory TNF-α production leading to caspase-8/3 mediated HKM apoptosis and the clearance of *M. fortuitum.* Our findings elucidate the role of mitochondria in innate immunity to *M. fortuitum*, which can be used for controlling mycobacteriosis (**Figure 6**).

## Data Availability Statement

The original contributions presented in the study are included in the article/**Supplementary Material**. Further inquiries can be directed to the corresponding author.

## Ethics Statement

Animal experiments performed in this study were approved by the Animal Ethics Committee, University of Delhi (DU/ZOOL/IAEC-R/2013/34), and the procedures were carried out according to the protocols approved by the Committee for the purpose of Control and Supervision of Experiments on Animals (CPCSEA), Government of India.

## Author Contributions

Conceived and designed the experiments: PD and SM. Performed the experiments: PD and MH. Analyzed the data: PD, MH, and SM. Contributed reagents/materials: SM. Wrote the paper: PD, MH, and SM. All authors contributed to the article and approved the submitted version.

## Funding

The research work was partially supported by Recurring Research Grant (RRG), South Asian University (RRG-2019), and DST-PURSE Grant University of Delhi (Government of India). The funders had no role in study design, data collection and analysis, decision to publish, or preparation of the manuscript.

## Conflict of Interest

The authors declare that the research was conducted in the absence of any commercial or financial relationships that could be construed as a potential conflict of interest.

## Publisher’s Note

All claims expressed in this article are solely those of the authors and do not necessarily represent those of their affiliated organizations, or those of the publisher, the editors and the reviewers. Any product that may be evaluated in this article, or claim that may be made by its manufacturer, is not guaranteed or endorsed by the publisher.

## Supplementary Material

Supplementary Figure 1
*M. fortuitum* induces mt-Ca^2+^ elevation. HKM (2 × 10^6^/mL) were infected with M*. fortuitum* and mt-Ca^2+^ levels measured at indicated time points p.i. using Rhod-2/AM. Individual assays were done in triplicates and the vertical bars represent mean ± SE of three independent observation (n=9). *^✽^P< 0.05* compared to HKM. HKM, uninfected HKM; HKM+B, HKM infected with *M. fortuitum.*

Supplementary Figure 2
*M. fortuitum* induces mtROS production. HKM (2 × 10^6^/mL) were infected with M*. fortuitum* and mtROS levels measured at indicated time points p.i. using MitoSOX. Individual assays were done in triplicates and the vertical bars represent mean ± SE of three independent observations (n=9). *^✽^P< 0.05* compared to HKM. HKM, uninfected HKM; HKM+B, HKM infected with *M. fortuitum*.

Supplementary Figure 3
*M. fortuitum* induces TNF-α production in HKM. **(A)** HKM (2 × 10^7^/mL) were infected with *M. fortuitum* and *TNF-α* expression quantified by RT-qPCR at indicated time points p.i. using SYBR green PCR master mix. **(B)** HKM (2 × 10^6^/mL) were infected with *M. fortuitum* and TNF-α production was quantified at indicated time points p.i. using specific assay kit. Individual assays were done in triplicates and the vertical bars represent mean ± SE of three independent observation (n=9). *^✽^P< 0.05* compared to HKM. HKM, uninfected HKM; HKM+B, HKM infected with *M. fortuitum.*
