# Supplementary figures and images for: mtROS Induced via TLR-2-SOCE Signaling Plays Proapoptotic and Bactericidal Role in Mycobacterium fortuitum-Infected Head Kidney Macrophages of Clarias gariepinus
Source: Front Immunol. 2021 Dec 20;12:748758. doi: 10.3389/fimmu.2021.748758 (PMC8720869; doi:10.3389/fimmu.2021.748758)

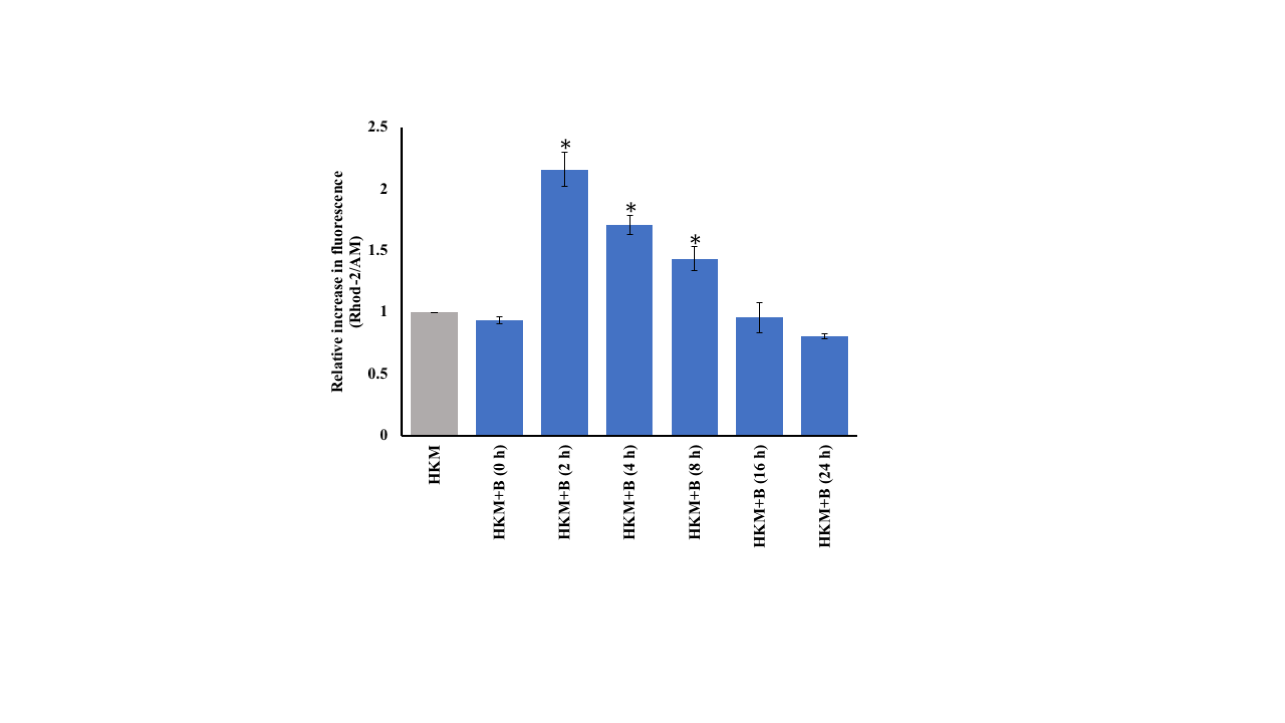

Supplement: Supplementary Figure 1 — M. fortuitum induces mt-Ca2+ elevation. HKM (2 × 10^6^/mL) were infected with M. fortuitum and mt-Ca2+ levels measured at indicated time points p.i. using Rhod-2/AM. Individual assays were done in triplicates and the vertical bars represent mean ± SE of three independent observation (n=9). ✽P< 0.05 compared to HKM. HKM, uninfected HKM; HKM+B, HKM infected with M. fortuitum. [file Image_1.tiff]

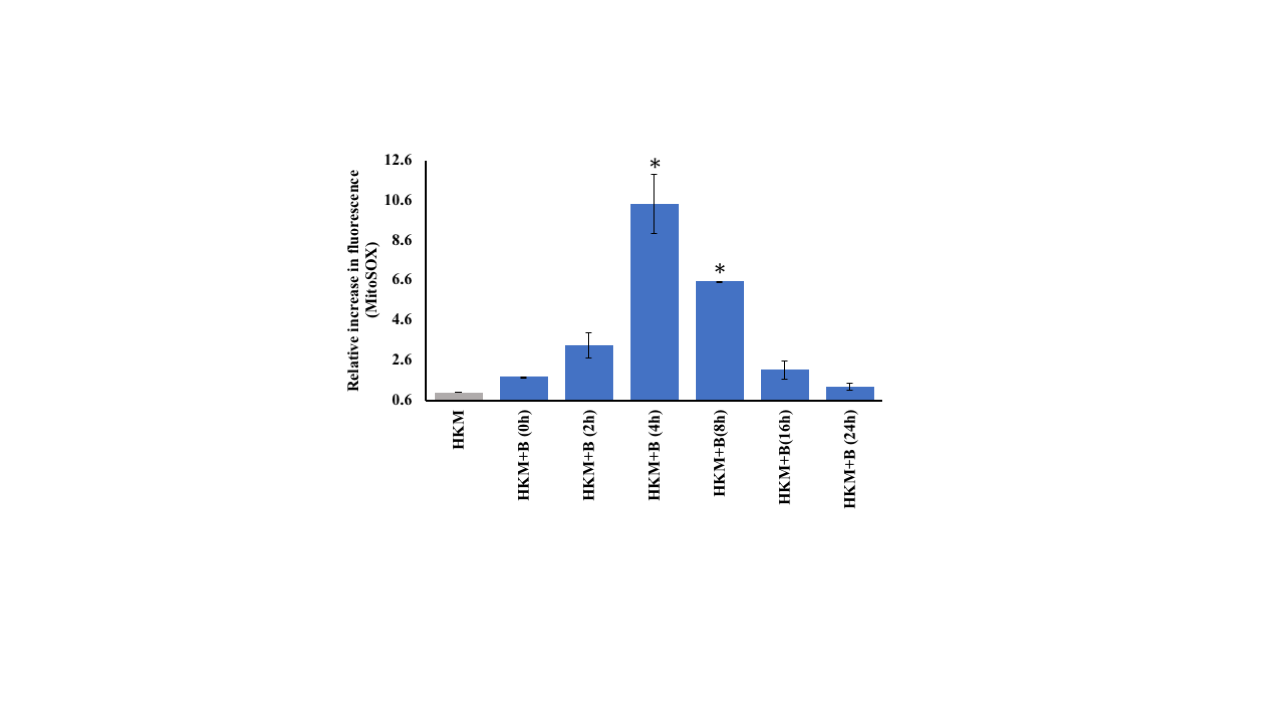

Supplement: Supplementary Figure 2 — M. fortuitum induces mtROS production. HKM (2 × 10^6^/mL) were infected with M. fortuitum and mtROS levels measured at indicated time points p.i. using MitoSOX. Individual assays were done in triplicates and the vertical bars represent mean ± SE of three independent observations (n=9). ✽P< 0.05 compared to HKM. HKM, uninfected HKM; HKM+B, HKM infected with M. fortuitum. [file Image_2.tiff]

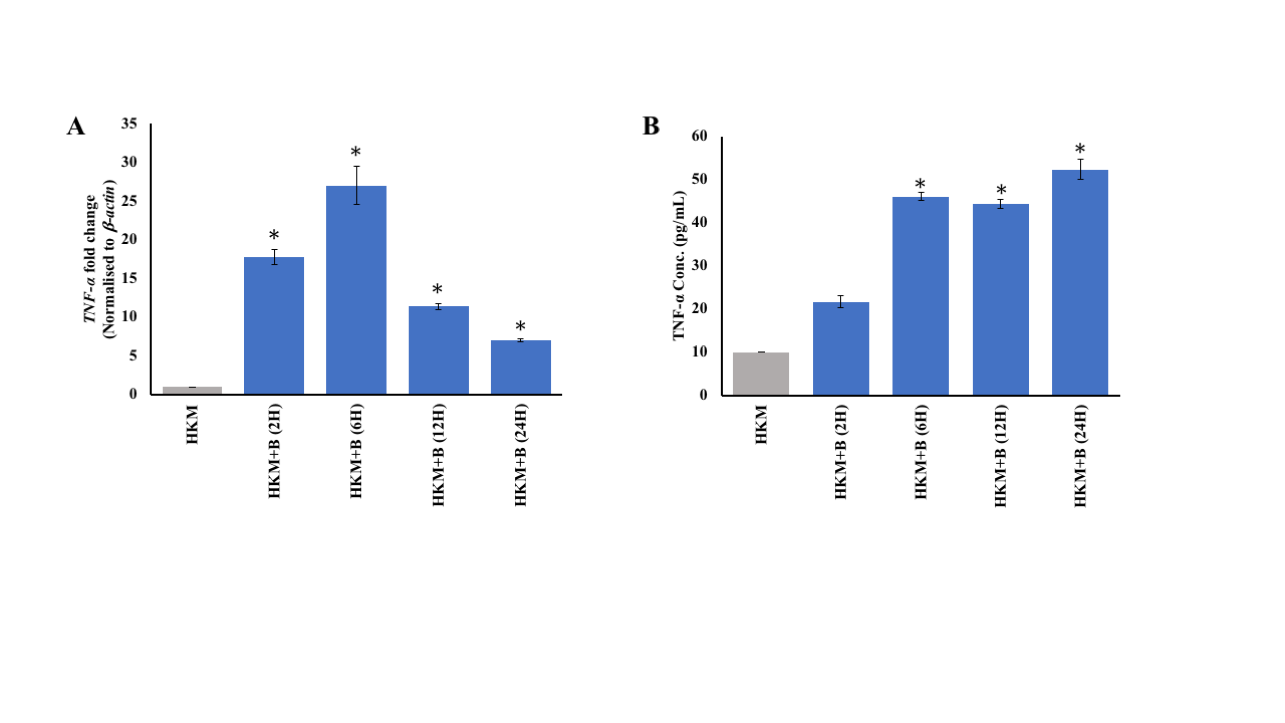

Supplement: Supplementary Figure 3 — M. fortuitum induces TNF-α production in HKM. (A) HKM (2 × 10^7^/mL) were infected with M. fortuitum and TNF-α expression quantified by RT-qPCR at indicated time points p.i. using SYBR green PCR master mix. (B) HKM (2 × 10^6^/mL) were infected with M. fortuitum and TNF-α production was quantified at indicated time points p.i. using specific assay kit. Individual assays were done in triplicates and the vertical bars represent mean ± SE of three independent observation (n=9). ✽P< 0.05 compared to HKM. HKM, uninfected HKM; HKM+B, HKM infected with M. fortuitum. [file Image_3.tiff]
